# Evaluation of surgical approach on reduction quality for transverse-family acetabulum fractures

**DOI:** 10.1186/s13018-025-06490-9

**Published:** 2025-12-09

**Authors:** Andrea Rincon, Erik-Matthew Sario, Aziz Saade, Chiara Giordani, Gillian L. Soles, Augustine M. Saiz

**Affiliations:** 1 University of California Davis School of Medicine, Sacramento, CA USA; 2 Department of Orthopaedic Surgery, University of California Davis, 4860 Y Street Suite 1700, Sacramento, CA 95817 USA; 3 California Northstate University College of Medicine, Elk Grove, CA USA

**Keywords:** Acetabulum, Fracture, Transverse, Reduction quality, Anterior approach, Posterior approach

## Abstract

**Introduction:**

Given the variety of transverse acetabular fracture patterns, different approaches are used for surgical fixation. The purpose of this study is to determine the association between surgical approach and fracture pattern on reduction quality in patients with transverse-family acetabular fractures.

**Methods:**

A retrospective review was performed on adult patients with transverse-family acetabular fractures undergoing surgical fixation at a level 1 trauma institution between 2017 and 2023. The primary outcome was reduction quality based on Matta’s criteria using postoperative computed tomography (CT) scans and X-rays. On CT scans, the greatest gap displacement and step-off was recorded in all three planes. On X-rays, gap displacement was measured on AP. Secondary outcomes included surgical complications.

**Results:**

On review, 47 patients were included, 68.1% were male with a mean age of 40.1, and an average follow-up of 8 months. Fractures included 12 transverse (approaches: 58.3% anterior, 25% posterior, and 16.7% percutaneous), 26 TP wall (88.4% posterior, 3.8% combined anterior-posterior (AP), and 7.8% percutaneous), and 10 T-shape (approaches: 50% anterior, 20% posterior, 10% combined AP, and 20% percutaneous). Reduction was assessed on postoperative CT with a significant difference in preoperative (6.82 ± 6.64 cm) and postoperative (1.96 ± 1.93 cm) mean maximal displacement (*p* < 0.001). On CT scans, 19% had anatomic reductions, 65% had good reductions, and 16% had poor reductions. There was no significant difference in CT or X-ray quality reduction between surgical approaches, transverse-family fracture type, hip dislocation on presentation, and surgeon experience, respectively. Patients who were cigarette smokers were more likely to have complications and Matta’s grade of ‘(*p* = 0.003, = 0.003, respectively).

**Conclusion:**

Although challenging, anatomic and good reductions can be achieved in transverse-family acetabular fractures. There were no observed differences in the quality of reduction or complication rates between different surgical approaches. Approaches for transverse-family acetabular fractures are predicated on careful fracture pattern assessment to determine surgical approach followed by technical execution.

## Introduction

Transverse-family acetabular fractures are relatively uncommon, affecting approximately 3 per 100,000 people annually in the United States [1, 2]. The incidence of these fractures displays a bimodal age distribution, primarily affecting young (< 40 years) individuals from high-energy trauma, or elderly adults from low-impact falls in the setting of decreased bone density [3, 4].

The underlying pathophysiology of transverse-family acetabular fractures involves the impact of the femoral head against the curved articular surface of the acetabulum; however, the pattern varies by position of the hip at the time of impact [4]. These fracture patterns are typically described using the Letournal and Judet classification system. This system categorizes acetabular fractures into five elementary and five associated types based on the involvement of the anterior and posterior columns and walls of the acetabulum. Transverse-family fractures, including transverse, transverse with posterior wall, and T-shaped types, are considered associated fracture because of the involvement of both acetabular columns through the dome [5–9]. The transverse component can be further subclassified based on location in relation to the weight-bearing dome as transtectal, juxtatectal, or infratectal. These variable fracture patterns contribute to the complexity, requiring thoughtful preop planning and surgical execution.

Most of these acetabular fractures require open reduction and internal fixation (ORIF); however, there is no consensus on a standard surgical approach based on fracture pattern. The most common surgical approaches include the modified ilioinguinal approach or the Kocher-Langenbeck approach (sometimes combined). While diverse fracture patterns warrant different approaches for repair, the lack of an approach algorithm complicates decision-making and may hinder optimal outcomes in the treatment of acetabular fractures.

Reduction of the articular surface and containment of the femoral head are critical for hip survivorship [1, 4]. Postoperative complications include posttraumatic arthritis and osteonecrosis, infection, iatrogenic nerve injury, deep vein thrombosis, and heterotopic ossification [4]. Different surgical approaches carry different risks profiles in these fractures.

The purpose of this study is to evaluate the association of surgical approaches for transverse-family acetabular fracture ORIF based fracture patterns and assess the resulting reduction quality. The secondary objective of this study is to identify factors contributing to malreduction and the incidence of postoperative complications.

## Patient and methods

Following Institutional Review Board approval, a retrospective study was conducted on patients who underwent surgery for transverse, transverse- posterior wall (TPW), and T-shaped acetabulum fractures at a single Level 1 trauma center over the 6-year period of 2017 to 2023. The T-shaped fractures were further classified based on the T-stem component in relation distally from the obturator foramen as anterior, posterior, or ischial. The transverse component for all subtypes was further classified into intratectal, juxtatectal, and transtectal. Inclusion criteria consisted of patients who underwent surgery for transverse-family acetabulum fractures who were 18 years of age or older and younger than 90 years of age at the time of surgery. The exclusion criteria included non-transverse-family acetabulum fractures, pregnant women, prisoners, and those with no preoperative and postoperative computed tomography (CT) and/or X-ray imaging. All surgeries were performed by three fellowship-trained orthopaedic trauma surgeons at our institution. Each surgeon independently selected the surgical approach based on the fracture pattern and intraoperative assessment. Surgeon experience was categorized as less than 5 years versus 5 or more years after fellowship. Electronic medical records were reviewed to obtain patient demographics, injury characteristics, surgery characteristics, postoperative follow-up complications, surgeon experience, mechanism of injury, pelvic injury and whether a hip dislocation was identified preoperatively.

Operative notes were reviewed to categorize what surgical approaches were used including anterior ilioinguinal and/or anterior intrapelvic approach (ANT), the Kocher-Langenbeck (KL) posterior approach, a combination of the two (ANT + KL), or percutaneous screw fixation. Fracture types were classified using Judet and Letournal by operating surgeon.

The anterior approach included the modified ilioinguinal and/or anterior intrapelvic (Stoppa) exposures, while the posterior approach used the standard Kocher-Langenbeck exposure. Representative illustrations and detailed descriptions of these approaches are available in previously published research (Cutreta et al. JAAOS 2015; Coughlin et al., Orthopaedics and Trauma 2018). These references are cited to guide readers unfamiliar with these surgical techniques [5, 7].

### Reduction quality

**Fig. 1:**
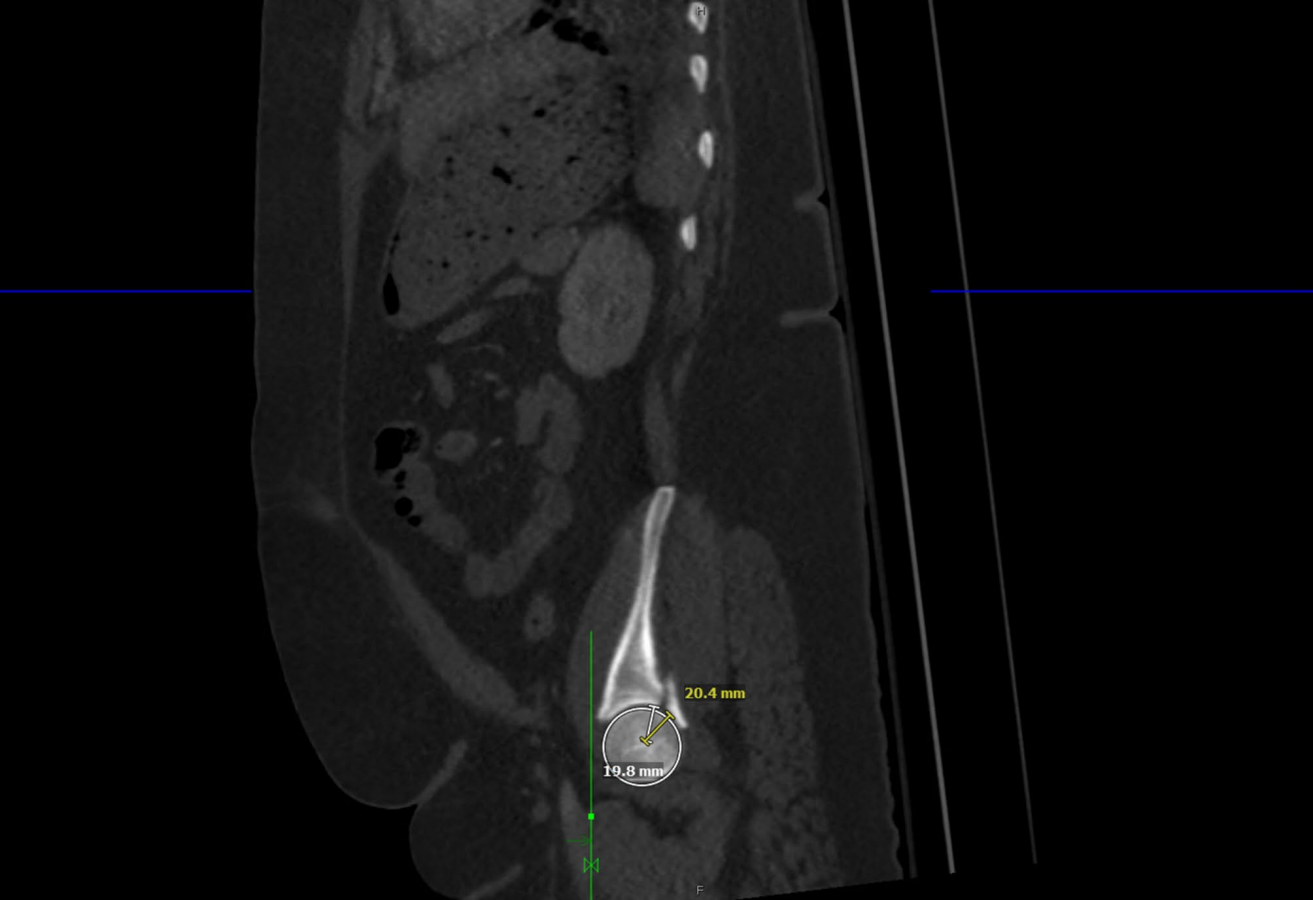
Step-off measurement in sagittal computed tomography axis

**Fig. 2:**
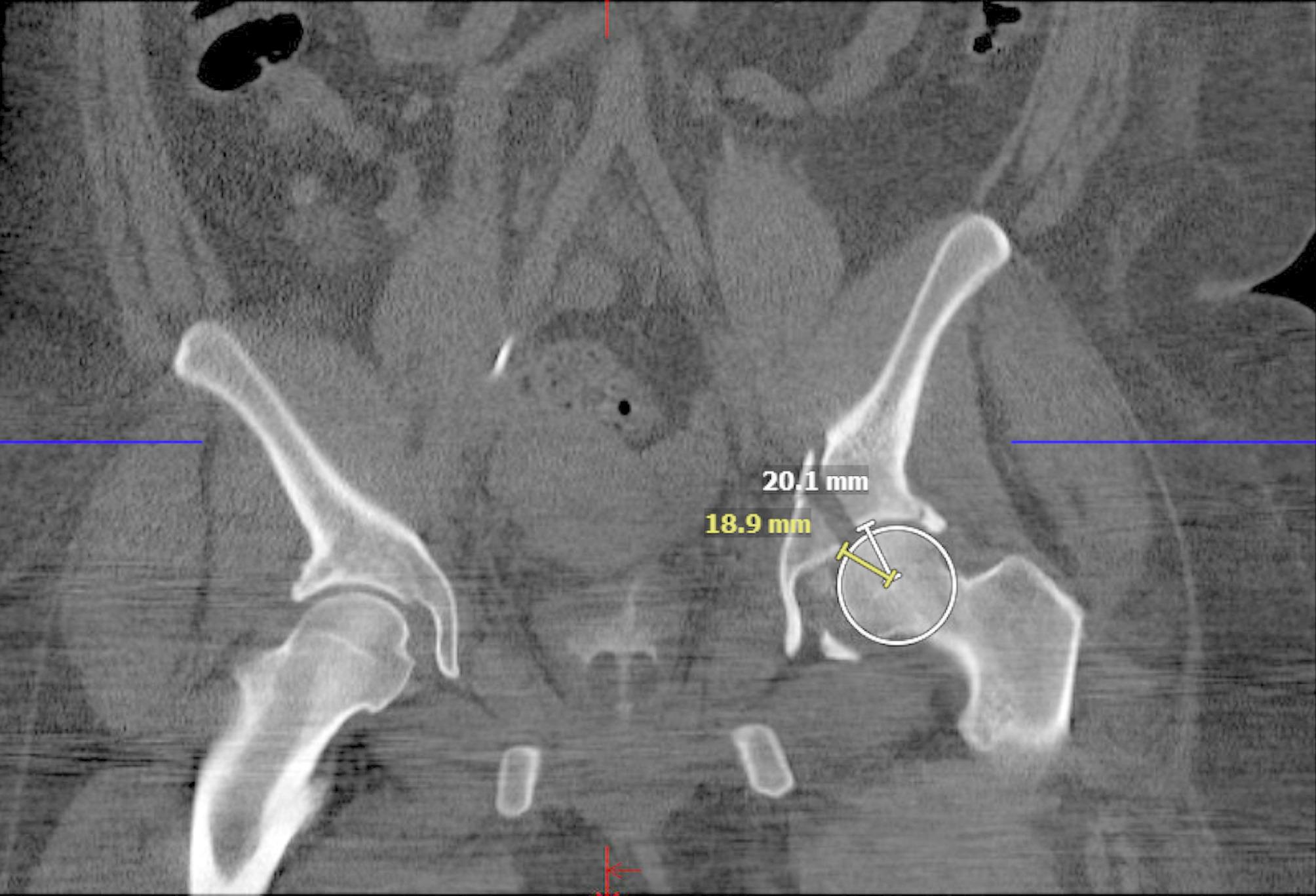
Step-off measurement in coronal computed tomography axis

**Fig. 3:**
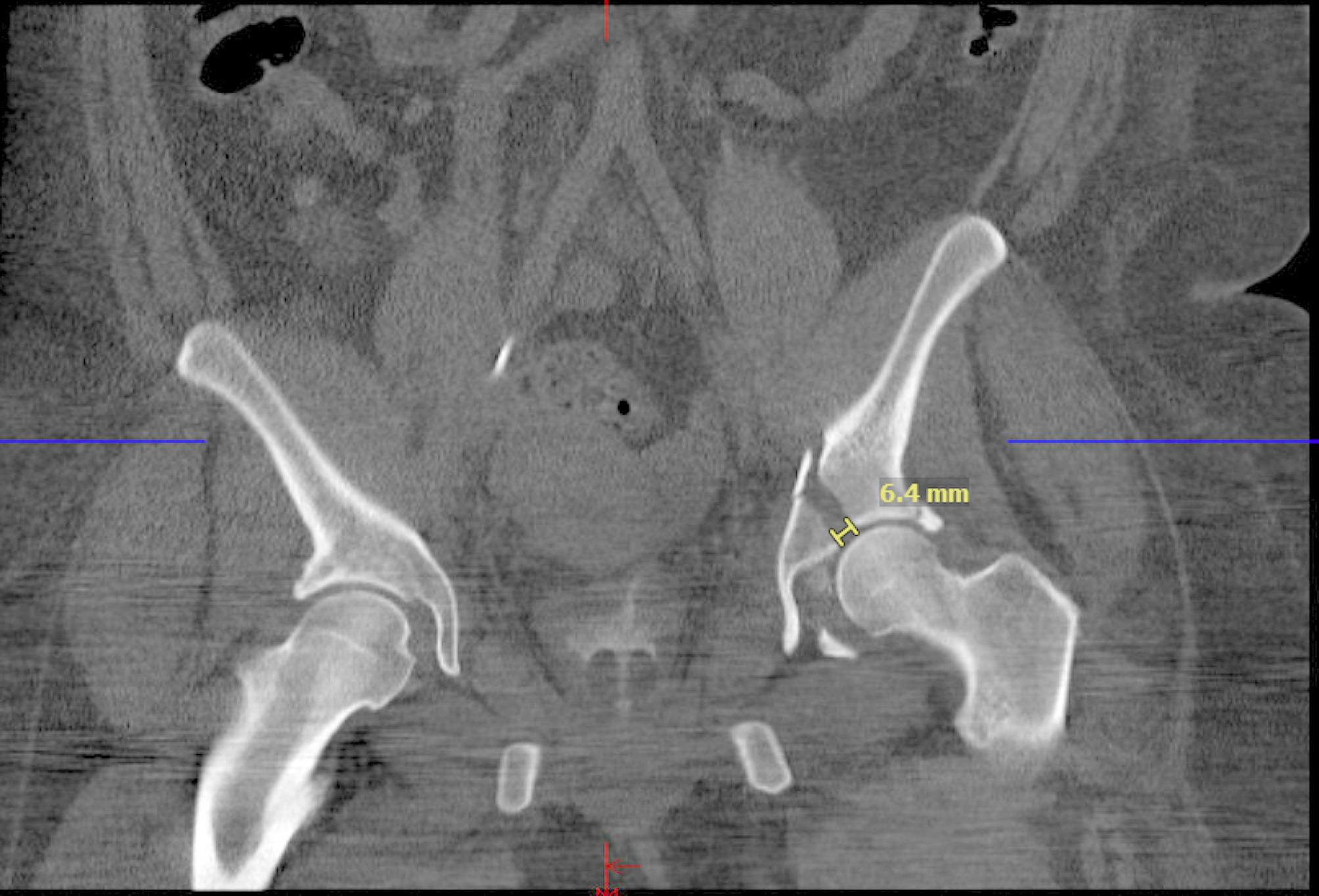
Max displacement of where the gap of the fracture is the largest in coronal computed tomography axis

All radiographic measurements were performed by a fellowship-trained orthopaedic trauma surgeon on CTs and radiographs. Initial displacement and postoperative reduction quality was determined by measuring maximal displacement and maximal step-off in axial, sagittal, and coronal on preoperative and postoperative CTs. Step-off measurements were completed by creating a circle to fit the acetabular dome and the center of that dome to the first point of step-off was measured using a ruler and a second line ruler started at that same center point to the second point of step-off, the difference between the two measurements was used as our main step-off measurement, a technique explained described by Verbeek et al. [10] (Figs. 1, 2 and 3).

X-ray max displacement was measured preoperatively and postoperatively using the posterior wall or the dome of the acetabulum. For both CT and X-ray, max displacement was measured using the largest space across the acetabular dome. Using Matta’s criteria, each measurement was classified as “excellent” (< 1 mm), “good” (1–3 mm), or “poor” (> 3 mm).

### Statistical analysis

Statistical analyses were conducted using Microsoft Excel (Microsoft Corporation, Redmond, WA). Data were first tested for normality using the Shapiro-Wilk test. For continuous variables that were normally distributed, independent samples t-tests were used to compare preoperative and postoperative measurements and were presented as mean differences with 95% confidence intervals (CI). For non-normally distributed data, the Mann-Whiteney U test was applied. Because most comparisons involved only two groups, ANOVA was not indicated. For comparisons involving more than two categories the data did not meet the assumptions of normality and homogeneity of variance required ANOVA; therefore, non-parametric methods were preferred. Chi-squared and Fischer’s exact tests were used to compare categorical variables. A *p*-value less than 0.05 was considered statistically significant.

## Results

In our review, 47 patients met inclusion criteria consisting of most commonly male (31/47) patients, with a mean age of 40.9 ± 19.1, and an average follow-up of 7.6 months. There were 12 transverse fractures with the following surgical approaches performed: 7 anterior, 3 posterior, and 2 percutaneous. There were 26 TPW fractures with the following surgical approaches performed: 23 posterior, 1 combined, and 2 percutaneous. There were 10 T-shape fractures with the following surgical approaches performed: 5 anterior, 2 posterior, 1 combined, and 2 percutaneous. The transverse fracture components were 31 transtectal, 14 juxtatectal and 3 infratectal (Table 1).

**Table 1:** Patient Demographics, fracture classification, and return to OR

Patient total, *n*	47^*^
Demographics	
Sex	
Male, n (%)	32, 68.0%
Female, n (%)	15, 32.0%
Average Age, years	40.9
T2DM, n (%)	4, 8.5%
BMI > 30, n (%)	22, 47.0%
BMI average	31
Cigarette use, n (%) Alcohol use, n (%)	6, 13.0% 2, 4.3%
Polytrauma, n (%)	34, 72.0%
*Fracture classification (Letournal), n*	**48**
Transverse, n (%)	12, 25.0%
Transverse + posterior wall, n (%)	26, 54.0%
T-shaped, n (%)	10, 21.0%
*Surgical Approach, n*	**48**
Anterior, n (%)	13, 27.0%
Posterior, n (%)	27, 56.0%
Combined, n (%)	2, 4.2%
Percutaneous, n (%)	6, 13.0%

BMI = Body mass index; OR = Operating room; T2DM = Type 2 diabetes mellitus

*One individual had two fractures, ultimately resulting in 48 fractures among 47 unique total patients

Reduction was noted to varying degrees with an overall preoperative mean maximal displacement (6.82 ± 6.64 cm) decreasing on postoperative CT (1.96 ± 1.93 cm) for each fracture (*p* < 0.001). On CT scans, 37/48 had excellent reductions, 10/48 had good reductions and 1/48 had poor reduction (Tables 2, 3).

**Table 2:** Outcomes based on demographics

	Complications			Matta’s Grading for Postoperative CT*			Matta’s Grading for Postoperative X-ray			
	Yes	No	*P*-value	Excellent	Good/Poor	*P*-value	Excellent	Good	Poor	*P*-value
Total	11	37	0.13	37	11	0.13	18	27	3	0.52
Sex (male)	8	25	0.75	25	8	0.75	14	17	2	0.57
T2DM	1	3	1.00	3	1	1.00	1	3	0	0.72
Cigarette use	5	3	0.003	3	5	0.003	5	3	0	0.28
Alcohol Use	1	1	0.41	1	1	0.41	0	2	0	0.57
Polytrauma	10	26	0.25	26	10	0.41	16	18	2	0.18
BMI			0.33			0.33				0.23
ASA			0.16			0.16				0.50

*Data analysis combined patients with “Good” or “Poor” matta’s scores for postoperative CT

* T2DM = Type 2 diabetes mellitus, BMI = body mass index, ASA = American society of anesthesiologist

**Table 3:** Quality of reduction

Measurement	Preoperative Mean (mm)	Preoperative SD (mm)	Postoperative Mean (mm)	Postoperative SD (mm)	*P*-value
CT - Axial	5.90	6.26	1.58	1.47	< 0.01
CT - Sagittal	4.60	5.80	1.72	1.94	
CT - Coronal	5.17	6.27	1.36	0.93	
CT - Highest Value	6.82	6.64	1.96	1.93	
XR Displacement	5.53	3.85	1.35	0.75	< 0.01

CT = Computed tomography scan; SD = Standard deviation; XR = X-ray

No statistical significance association was identified between surgical approach and the type of transverse fracture component: transtectal, juxtatectal, or infratectal (*p* = 0.399; Table 6).

There was no significant difference in CT or X-ray quality reduction between surgical approaches(*p* = 0.71; *p* = 0.06), transverse fracture component location (transtectal, juxtatectal, and infratectal) (*p* = 0.78; *p* = 0.43), fracture type (*p* = 0.65; *p* = 0.19), hip dislocation on presentation (*p* = 0.20; *p* = 0.44), and surgeon experience (*p* = 0.88; *p* = 0.10), respectively (Tables 4 and 5). No significant differences between surgeon experience suggests consistent outcomes across surgeons and supports the generalizability of these findings. One patient had bilateral fractures, computing a total of 48 fractures included in the analysis. No significant differences were determined for Matta’s grades for postoperative CT based on age, sex, diabetic status, alcohol use, BMI, polytrauma status, or ASA. However, zero of the 11 patients who reported a history of smoking had an “excellent” Matta’s grade (*p* = 0.003) on CT. Matta’s grades for postoperative X-ray were not significantly different across age, sex, diabetic status, smoking history, alcohol use, BMI, polytrauma status, orASA.

**Table 4:** Matta Grading for postoperative CT

		Excellent	Good	Poor	Total
Surgical approach	Anterior	3	8	2	13
	Posterior	4	18	5	27
	Combined	0	1	1	2
	Percutaneous	2	4	0	6
	Total	9	31	8	48
	*P*-value	0.71			
Transverse fracture to acetabular roof relationship	Transtectal	5	20	6	31
	Juxtatectal	4	8	2	14
	Infratectal	0	3	0	3
	Total	9	31	8	48
	*P*-value	0.78			
Transverse-family fracture classification	Transverse	4	6	2	12
	Transverse-PW	4	18	4	26
	T-shaped	1	7	2	10
	Total	9	31	8	48
	*P*-value	0.65			
Surgeon experience, years	< 5	4	7	0	11
	> 5	7	23	7	37
	*P*-value	0.88			
Hip dislocation	Dislocation	2	16	5	23
	No dislocation	7	15	3	25
	*P*-value	0.20			

Trans-PW = Transverse posterior wall

**Table 5:** Matta grading for postoperative X-ray

		Excellent	Good	Poor	Total
Surgical approach	Anterior	7	5	0	12
	Posterior	7	18	1	26
	Combined	1	0	1	2
	Percutaneous	2	3	1	6
	Total	17	26	3	46
	*P*-value	0.06			
Transverse fracture to acetabular roof relationship	Transtectal	11	18	1	30
	Juxtatectal	5	7	1	13
	Infratectal	1	1	1	3
	Total	17	26	3	46
	*P*-value	0.43			
Transverse-family fracture classification	Transverse	7	4	0	11
	Trans-PW	6	17	2	25
	T-shaped	4	5	1	10
	Total	17	26	3	46
	*P*-value	0.19			
Surgeon experience, years	< 5	5	6	0	11
	> 5	16	18	3	37
	*P*-value	0.44			
Hip dislocation	Dislocation	6	14	2	22
	No dislocation	11	12	1	24
	*P*-value	0.44			

Trans-PW = Transverse posterior wall

*Pre-/postoperative comparisons could not be performed for two patients as one patient did not receive a postoperative X-ray while another did not receive a preoperative X-ray. These fractures were excluded from this portion resulting in 46 considered fractures

Complications occurred in 12 of the 48 procedures: 3 DVTs, 1 sciatic nerve palsy with the Kocher-Langenbeck approach, and 1 patient with sepsis. Of the 48 fractures, 7 (transverse (2), TPW (3), T-shape (2)) returned to the OR (3 anterior approach, 3 posterior approach, 1 combined approach, and 0 percutaneous approach) for revision surgeries (3 total hip arthroplasty, 3 debridement for deep infection, and 1 implant removal). The quality reduction in the three patients who underwent subsequent THA was assessed by postoperative imaging. Postoperative computed tomography (CT) demonstrated good reduction in two cases and poor reduction in one case. Postoperative radiographs showed excellent reduction in one case and good reduction in two cases. The intervals from initial open reduction and internal fixation (ORIF) to THA were 3 days, 11 months, and undetermined for the third patient. For the latter case, the patient’s medical record contained a gap from 2018 to 2023; however, a CT scan obtained in September 2023 confirmed that a THA had been performed during this interval (Table 6).

**Table 6:** Comparison of surgical approaches by transverse component location (transtectal, juxtatectal, infratectal)

		Transverse Component Location			
		Transtectal	Juxtatectal	Infratectal	P-value
Surgical Approach	Anterior	7	5	0	0.399
	Posterior	20	6	2	
	Combined	2	0	0	
	Percutaneous	2	3	1	

Complication rates were not significantly different between surgical approaches (*p* = 0.20) (Table 2). Furthermore, no significant difference in complication rates was observed based on age, sex, diabetic status, alcohol use, BMI, polytrauma status, or ASA. Patients who reported cigarette smoking, however, were more likely to experience complications following the procedure, with 5 of the 11 patients who experienced complications reporting a history of smoking (*p* = 0.003).

## Discussion

To our knowledge, this is the largest study to assess reduction quality in transverse-family acetabulum fractures when compared to surgical approaches. Given the variety of transverse family patterns and characteristics of subtypes, different surgical approaches may be used. Although some risk factors for poor surgical outcomes have been identified [11], further study is warranted, and additional factors remain to be established. No statistically significant association was observed between surgical approach and the location of the transverse component (*p* = 0.399), suggesting that a more nuanced approach to fracture morphology may influence the choice ofapproach.

In addition, outcomes in acetabular fracture surgery are influenced by numerous patient and injury-related variables, including patient age, mechanism of injury, bone quality, and the presence of associated injuries or comorbidities. These factors contribute to the heterogeneity of this patient population, underscoring the inherent complexity of both pelvic surgery and research in this field. Accounting for these variables is essential when interpreting results and comparing studies across institutions.

Our findings share some similarities to a retrospective study by Kim et al. who assessed various surgical approaches and outcomes for T-shape acetabular fractures [12]. In this retrospective study, they evaluated the reduction quality using Matta’s criteria after different surgical interventions, including anterior intrapelvic approach (AIP), posterior approach (KL), and percutaneous screw fixation. They concluded that the surgical approach to T-shape acetabular fractures does not impact reduction quality on postoperative CT or the postoperative complication rates. Poor reductions on CT (> 3 mm) were also not associated with fracture classification, unstable pelvic ring injuries, posterior wall fractures, T-stem component, transverse component, preoperative articular displacement, preoperative femoral head protrusion, or surgeon experience. Their finding that 50% of all patients in the study experienced complications underscores the complexity of this fracture type and the challenges in achieving satisfactory outcomes regardless of surgical approach.

Comparable findings have been reported in broader series that included a high proportion of transverse and transverse-posterior wall fractures. For example, Matta and Tornetta 2010 evaluated acetabular fractures treated through the ilioinguinal approach over ten years and found that surgical approach selection, when guided by fracture morphology and surgeon experience, did not significantly influence reduction quality or complication rates [6]. These results align without findings that outcomes were not dependent on approach but rather on technical execution and appropriate surgical preoperative planning. In Jang et al.’s study investigating prognostic factors associated with poor surgical outcomes for transverse acetabular fractures, multivariate analysis identified dome impaction and wide residual gaps as significant risk factors. In patients with residual gap and step > 3 mm and 1 mm respectively, the development of osteoarthritis increased significantly. Additional risk factors identified for poor outcomes include age over 50 years, BMI classification of overweight, high-energy trauma, articular surface communication or impaction, large initial displacement, femoral head dislocation, residual gap, residual step, and surgeon training [13]. When using axial, coronal, and sagittal CT views to evaluate reduction quality according to Matta’s criteria and implant positions 48–72 h after surgery, the authors identified a relatively low rate of satisfactory reduction. A major limitation of this study was the small sample size, so the authors concluded that a larger study with additional variables should be carried out to confirm their results. Our results demonstrated that patients who identified as cigarette smokers were more likely to encounter a complication versus those who did not smoke. In addition, these same patients were more likely to have a Matta’s grade of poor. Otherwise, medical comorbidities were not associated with worse outcomes or increased complications.

Similar studies such as Hu et al. (2017) investigated the surgical treatment of transverse acetabular fractures with or without posterior wall involvement. In this study, 7 pure transverse and 14 with posterior wall fractures of the acetabulum were surgically treated with anterior column fractures first reduced and temporarily fixed *via* a modified Smith-Peterson incision before final fixation following posterior column and wall reduction *via* a Kocher-Langenbeck approach. They found that this approach successfully resulted in anatomic reduction of the anterior column in 20/21 cases and the posterior column in all cases [14]. After following up 18 cases over an average of 16.3 months, they found osseous union in all fractures. Complications included avascular necrosis of the femoral head in 1 case, heterotopic ossification in 3 cases, and anterolateral thigh numbness in 6 cases. Based on these results, the authors recommended sequential reduction and fixation of anterior and posterior columns. They suggested modified Smith-Peterson incision for management of the anterior column is advantageous for direct visualization and to minimize invasiveness. This study has several strengths, including the granular detail obtained for each patient, such as fracture classification, surgical approach, complications, reduction quality, and patient demographics. Most patients underwent CT imaging, allowing for thorough fracture assessment and postoperative evaluation. The study also incorporated a structured model assessment to analyze key variables. However, there were several limitations. The total sample size, as well as the sample size for each surgical approach, was limited. The retrospective nature of the study introduced potential biases and data collection constraints. While no significant differences were observed in surgical approach or reduction quality, patient selection may have influenced the primary outcome. Lastly, patient loss to follow-up restricted the amount of postoperative data available for analysis.

The absence of functional outcome or mobility data represents a significant limitation of this study. While radiographic outcomes and complication rates provide objective measures of surgical success, they do not fully capture the patient’s functional recovery or satisfaction. Prior studies have demonstrated that radiographic reduction does not always correlate with patient reported outcomes, including pain, mobility, and return to activity. Future investigations should incorporate validated functional scoring systems, such as the Harris Hip Score of the Merle d’ Aubigné and Postel score, to correlate radiographic findings with clinical outcomes better [4, 11].

While this study primary focused on comparative analyses between surgical approaches, correlation or regression analyses examining associations between radiographic outcomes and clinical parameters, such as age, mechanism of injury, or comorbidities, were not performed. Future studies incorporating these analyses could provide additional insight into predictors of reduction quality, complications, and long-term functional outcomes in transverse-family acetabular fractures.

## Conclusion

While challenging, anatomic and good reductions can be achieved in transverse-family acetabular fractures. This retrospective chart review found no significant difference in the quality of reduction or complication rates across different surgical approaches. Approaches for transverse-family acetabular fractures are predicated on careful fracture pattern assessment and technical execution. No difference in approach or outcomes was observed based on location of the transverse fracture component—transtectal, juxtatectal, or infratectal. This finding suggests that other more nuanced fracture morphology characteristics may influence approach selection. Further studies may seek to elucidate how specific fracture characteristics guide operative decision-making in these complex injuries.

## Data Availability

No datasets were generated or analysed during the current study.

## References

[1] Hoge S, Chauvin BJ. Acetabular fractures. StatPearls [Internet]. Treasure Island (FL): StatPearls Publishing; 2024. 31335035

[2] Singh A, Telagareddy K, Kumar P, Singh S, Singh RN, Singh PK. THA in patients with neglected acetabular fractures. SICOT-J. 2022;8:37. 36040232 10.1051/sicotj/2022028 PMC9426301

[3] Toro G, Braile A, De Cicco A, et al. Fragility fractures of the acetabulum: current concepts for improving outcomes. J Orthop Traumatol. 2022;56(7):1139–49. 10.1007/s43465-022-00653-0 PMC9232661 35813545

[4] Ziran N, Soles GLS, Matta JM. Outcomes after surgical treatment of acetabular fractures: a review. Patient Saf Surg. 2019;13:16. 30923570 10.1186/s13037-019-0196-2 PMC6420740

[5] Cutrera NJ, Pinkas D, Toro JB. Surgical approaches to the acetabulum and modifications in technique. J Am Acad Orthop Surg. 2015;23(10):592–603. 26320164 10.5435/JAAOS-D-14-00307

[6] Matta JM, Tornetta P III. Surgical treatment of acetabular fractures through the ilioinguinal approach: a 10-year perspective. Injury. 2010;41(8):839–41.20451195

[7] Coughlin TA, Shivji FS, Quah C, Forward DP. Acetabular fractures, anatomy and implications for treatment. Orthop Trauma. 2018;32(2):116–20.

[8] Alton TB, Gee AO. Classifications in brief: letournel classification for acetabular fractures. Clin Orthop Relat Res. 2014;472(1):35–8. 10.1007/s11999-013-3375-y. 24214824 10.1007/s11999-013-3375-y PMC3889427

[9] Judet R, Judet J, Letournel E. Fractures of the acetabulum: classification and surgical approaches for open reduction. J Bone Joint Surg Am. 1964;46:1615–46. 14239854

[10] Verbeek DO, Van Der List JP, Moloney GB, Wellman DS, Helfet DL. Assessing postoperative reduction after acetabular fracture surgery: a CT-based method. J Orthop Trauma. 2018;32(7):e284–8. 29481491 10.1097/BOT.0000000000001161

[11] Jang JH, Moon NH, Rhee SJ, Jung SJ, Ahn TY. Surgical outcomes of transverse acetabular fractures and risk factors for poor outcomes. BMC Musculoskelet Disord. 2021;22:222. 33648482 10.1186/s12891-021-04082-2 PMC7923451

[12] Kim YJ, Foodoul MA, Parry JA, Mauffrey C. Surgical approach of T-type acetabular fractures and postoperative complications. Injury. 2023;54(6):1687–92. 10.1016/j.injury.2023.03.037 37059600

[13] Matta JM. Fractures of the acetabulum: accuracy of reduction and clinical results. J Bone Joint Surg Am. 1996;78(11):1632–45. 8934477

[14] Hu T, Xu H, Jiang C, Ren G, An Z. Treatment of transverse with or without posterior wall fractures of acetabulum using a modified Smith-Petersen combined with Kocher-Langenbeck approach. Med Sci Monit. 2017;23:2765–74. 28588152 10.12659/MSM.901966 PMC5470865

